# *N*,*N*′-Dicyclo­hexyl-*N*′′-(4-nitro­benzo­yl)phospho­ric triamide

**DOI:** 10.1107/S1600536810000851

**Published:** 2010-01-13

**Authors:** Fahimeh Sabbaghi, Mehrdad Pourayoubi, Maryam Toghraee, Vladimir Divjakovic

**Affiliations:** a Department of Chemistry, Islamic Azad University–Zanjan Branch, PO Box 49195-467, Zanjan, Iran; b Department of Chemistry, Ferdowsi University of Mashhad, Mashhad 91779, Iran; c Department of Physics, Faculty of Sciences, University of Novi Sad, Trg D. Obradovica 3, 21000 Novi Sad, Serbia

## Abstract

The P atom in the title compound, C_19_H_29_N_4_O_4_P, exhibits a tetra­hedral coordination and the phosphoryl and carbonyl groups are *anti* to each other. Adjacent mol­ecules are linked by N—H⋯O hydrogen bonds to form a layer motif.

## Related literature

For a phosphate compound containing the C(O)NHP(O) unit, see: Pourayoubi & Sabbaghi (2007 ▶). For phosphoric triamide, see: Pourayoubi & Sabbaghi (2009 ▶).
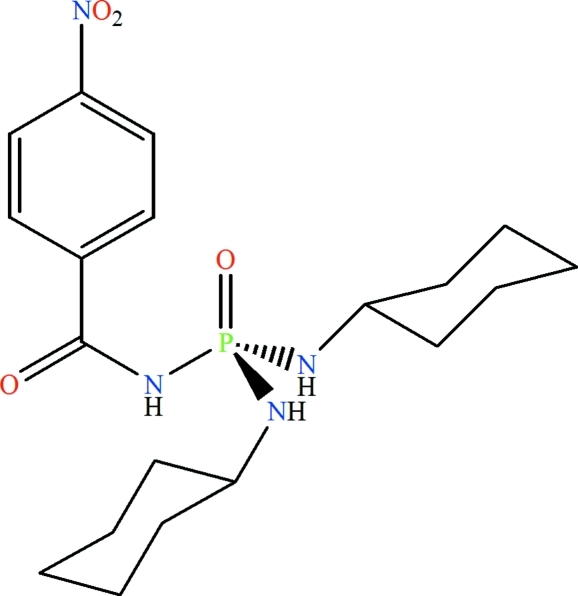


## Experimental

### 

#### Crystal data

C_19_H_29_N_4_O_4_P
*M* *_r_* = 408.43
Triclinic, 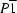

*a* = 10.4091 (7) Å
*b* = 10.8527 (9) Å
*c* = 11.1116 (10) Å
α = 99.764 (7)°
β = 110.881 (7)°
γ = 108.158 (7)°
*V* = 1057.25 (18) Å^3^
*Z* = 2
Mo *K*α radiation
μ = 0.16 mm^−1^
*T* = 295 K
0.52 × 0.31 × 0.29 mm

#### Data collection

Oxford Diffraction Xcalibur diffractometer with a Sapphire3 (Gemini Mo) detector
6746 measured reflections
3713 independent reflections
2915 reflections with *I* > 2σ(*I*)
*R*_int_ = 0.016

#### Refinement

*R*[*F* ^2^ > 2σ(*F* ^2^)] = 0.040
*wR*(*F* ^2^) = 0.115
*S* = 1.11
3713 reflections
254 parameters
H-atom parameters constrained
Δρ_max_ = 0.38 e Å^−3^
Δρ_min_ = −0.37 e Å^−3^

### 

Data collection: *CrysAlis PRO* (Oxford Diffraction, 2009 ▶); cell refinement: *CrysAlis PRO*; data reduction: *CrysAlis PRO*; program(s) used to solve structure: *SIR92* (Altomare *et al.*, 1993 ▶); program(s) used to refine structure: *SHELXL97* (Sheldrick, 2008 ▶); molecular graphics: *Mercury* (Macrae *et al.*, 2008 ▶); software used to prepare material for publication: *SHELXL97*.

## Supplementary Material

Crystal structure: contains datablocks I, global. DOI: 10.1107/S1600536810000851/ng2716sup1.cif

Structure factors: contains datablocks I. DOI: 10.1107/S1600536810000851/ng2716Isup2.hkl

Additional supplementary materials:  crystallographic information; 3D view; checkCIF report

## Figures and Tables

**Table 1:** Hydrogen-bond geometry (Å, °)

*D*—H⋯*A*	*D*—H	H⋯*A*	*D*⋯*A*	*D*—H⋯*A*
N1—H1⋯O4^i^	0.86	2.54	3.305 (2)	148
N2—H2⋯O2^ii^	0.86	2.25	3.0578 (18)	156
N3—H3⋯O1^iii^	0.86	1.97	2.8229 (18)	170

Symmetry codes: (i) 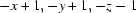; (ii) 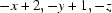; (iii) 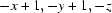.

**Fig. 1.**
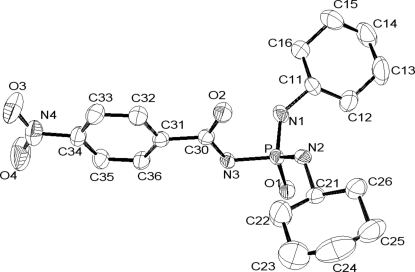
The molecular structure of the title compound, indicating the atom labeling scheme. Displacement ellipsoids are drawn at the 50% probability level, the H atoms were omitted for clarity.

**Fig. 2.**
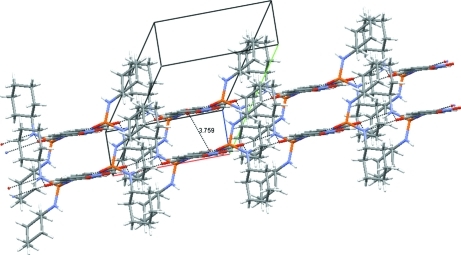
Part of the crystal structure with hydrogen bonds shown as dashed lines and the centroid of phenyl rings as ball representation.

## supplementary crystallographic information

### Comment

Following our previous works about phosphorus compounds containing C(O)NHP(O) moiety such as P(O)[NHC(O)C_6_H_4_(4-NO_2_)][N(CH(CH_3_)_2_)(CH_2_C_6_H_5_)]_2_ (Pourayoubi & Sabbaghi, 2009) and [(C_6_H_5_CH_2_)(CH(CH_3_)_2_)NH_2_][CCl_3_C(O)NHP(O)(O)(OCH_3_)] (Pourayoubi & Sabbaghi, 2007), we report here on the synthesis and crystal structure of a new phosphaza-analogous of β-diketone, P(O)[NHC(O)C_6_H_4_(4-NO_2_)][NHC_6_H_11_]_2_. Single crystals of title compound were obtained from a solution of CH_3_CN and CH_3_OH after slow evaporation at room temperature. The phosphoryl and the carbonyl groups are *anti* and the phosphorus atom has a slightly distorted tetrahedral configuration (Fig. 1). The bond angles around the P atom are in the range of 101.89 (8)°-119.46 (8)°. The P—N3 bond length (1.6966 (14) Å) is longer than the P—N1 and P—N2 bond lengths (1.6174 (16) Å and 1.6233 (14) Å). In the crystal network of title compound, P(O)[NHC(O)C_6_H_4_(4-NO_2_)][NHC_6_H_11_]_2_, molecules are linked *via* P═O···H—N (O1···N3 = 2.8229 (18) Å) and C═O···H—N (O2···N2 = 3.0578 (18) Å) hydrogen bonds in the linear arrangement along *a* axis. Moreover, molecules are aggregated through the weak hydrogen bonds O_nitro_···H—N (O4···N1 = 3.305 (2) Å) parallel to the *c* axis and π–π stacking interactions between neighboring 4-NO_2_—C_6_H_4_—C(O)NH– moieties [centroid–centroid distance = 3.759 (1) Å], Fig. 2.

### Experimental

The reaction of phosphorus pentachloride (4.165 g, 20 mmol) and 4-nitrobenzamide (3.323 g, 20 mmol) in dry CCl_4_ (70 ml) at 353 K (3 h) and then the treatment of formic acid (0.921 g, 20 mmol) at room temperature leads to 4-NO_2_—C_6_H_4_C(O)NHP(O)Cl_2_. The solid (4-NO_2_—C_6_H_4_C(O)NHP(O)Cl_2_) was washed with dry CCl_4_. To a solution of (0.708 g, 2.5 mmol) 4-NO_2_—C_6_H_4_C(O)NHP(O)Cl_2_ in CH_3_CN (40 ml), a solution of cyclohexylamine (0.992 g, 10 mmol) in CH_3_CN (10 ml) was added dropwise at 273 K. After 6 h of stirring, the solvent was evaporated in vacuum. The solid was washed with distilled water. Single crystals were obtained from a solution of the title compound in CH_3_CN and CH_3_OH after slow evaporation at room temperature. IR (KBr, cm^-1^): 3056, 2922, 2870, 2770, 2689, 2641, 2589, 2489, 2412, 2169, 2007, 1954, 1678, 1602, 1515, 1446, 1344, 1230, 1039, 963, 860, 736, 702.

### Refinement

H atoms were placed in the calculated positions and included in the refinement in a riding-model approximation with C—H = 0.93–0.98 Å, N—H = 0.86Å and *U*_iso_(H) = 1.2*_U_*_eq_(C,*N*).

### Crystal data

**Table:**

C_19_H_29_N_4_O_4_P	*Z* = 2
*M**_r_* = 408.43	*F*(000) = 436
Triclinic, *P*1	*D*_x_ = 1.283 Mg m^−^^3^
Hall symbol: -P 1	Mo *K*α radiation, λ = 0.71073 Å
*a* = 10.4091 (7) Å	Cell parameters from 4113 reflections
*b* = 10.8527 (9) Å	θ = 3.3–29.2°
*c* = 11.1116 (10) Å	µ = 0.16 mm^−^^1^
α = 99.764 (7)°	*T* = 295 K
β = 110.881 (7)°	Prism, colorles
γ = 108.158 (7)°	0.52 × 0.31 × 0.28 mm
*V* = 1057.25 (18) Å^3^	

### Data collection

**Table:**

Oxford Diffraction Xcalibur diffractometer with a Sapphire3 (Gemini Mo) detector	2915 reflections with *I* > 2σ(*I*)
Radiation source: Enhance (Mo) X-ray Source	*R*_int_ = 0.016
graphite	θ_max_ = 25.0°, θ_min_ = 3.3°
Detector resolution: 16.3280 pixels mm^-1^	*h* = −12→12
ω scans	*k* = −12→11
6746 measured reflections	*l* = −11→13
3713 independent reflections	

### Refinement

**Table:**

Refinement on *F*^2^	Secondary atom site location: difference Fourier map
Least-squares matrix: full	Hydrogen site location: inferred from neighbouring sites
*R*[*F*^2^ > 2σ(*F*^2^)] = 0.040	H-atom parameters constrained
*wR*(*F*^2^) = 0.115	*w* = 1/[σ^2^(*F*_o_^2^) + (0.068*P*)^2^] where *P* = (*F*_o_^2^ + 2*F*_c_^2^)/3
*S* = 1.11	(Δ/σ)_max_ = 0.001
3713 reflections	Δρ_max_ = 0.38 e Å^−^^3^
254 parameters	Δρ_min_ = −0.37 e Å^−^^3^
0 restraints	Extinction correction: *SHELXL97* (Sheldrick, 2008), Fc^*^=kFc[1+0.001xFc^2^λ^3^/sin(2θ)]^-1/4^
Primary atom site location: structure-invariant direct methods	Extinction coefficient: 0.015 (3)

### Special details

**Table:**

Experimental. #__ type_ start__ end____ width___ exp.time_ 1 omega -7.00 53.00 1.0000 3.6500 omega____ theta____ kappa____ phi______ frames - 21.1985 77.0000 150.0000 60#__ type_ start__ end____ width___ exp.time_ 2 omega -4.00 91.00 1.0000 3.6500 omega____ theta____ kappa____ phi______ frames - 21.1985 77.0000 30.0000 95#__ type_ start__ end____ width___ exp.time_ 3 omega -51.00 47.00 1.0000 3.6500 omega____ theta____ kappa____ phi______ frames - 21.1985 - 37.0000 240.0000 98#__ type_ start__ end____ width___ exp.time_ 4 omega -51.00 34.00 1.0000 3.6500 omega____ theta____ kappa____ phi______ frames - 21.1985 - 37.0000 150.0000 85#__ type_ start__ end____ width___ exp.time_ 5 omega -6.00 33.00 1.0000 3.6500 omega____ theta____ kappa____ phi______ frames - 21.1985 77.0000 270.0000 39
Geometry. All e.s.d.'s (except the e.s.d. in the dihedral angle between two l.s. planes) are estimated using the full covariance matrix. The cell e.s.d.'s are taken into account individually in the estimation of e.s.d.'s in distances, angles and torsion angles; correlations between e.s.d.'s in cell parameters are only used when they are defined by crystal symmetry. An approximate (isotropic) treatment of cell e.s.d.'s is used for estimating e.s.d.'s involving l.s. planes.
Refinement. Refinement of *F*^2^ against ALL reflections. The weighted *R*-factor *wR* and goodness of fit *S* are based on *F*^2^, conventional *R*-factors *R* are based on *F*, with *F* set to zero for negative *F*^2^. The threshold expression of *F*^2^ > σ(*F*^2^) is used only for calculating *R*-factors(gt) *etc*. and is not relevant to the choice of reflections for refinement. *R*-factors based on *F*^2^ are statistically about twice as large as those based on *F*, and *R*- factors based on ALL data will be even larger.

### Fractional atomic coordinates and isotropic or equivalent isotropic displacement parameters (Å^2^)

**Table:**

	*x*	*y*	*z*	*U*_iso_*/*U*_eq_	
P	0.69819 (5)	0.43097 (5)	0.04401 (5)	0.03153 (17)	
O1	0.59696 (13)	0.43337 (14)	0.10845 (13)	0.0427 (3)	
O2	0.86256 (14)	0.52969 (15)	−0.11240 (13)	0.0494 (4)	
O3	0.6421 (2)	0.7685 (2)	−0.64611 (17)	0.0824 (6)	
O4	0.4469 (2)	0.7627 (2)	−0.62270 (19)	0.0973 (7)	
N1	0.67651 (16)	0.28654 (15)	−0.04742 (16)	0.0416 (4)	
H1	0.6103	0.2583	−0.1305	0.050*	
N2	0.87293 (15)	0.49971 (15)	0.15558 (14)	0.0337 (4)	
H2	0.9336	0.4661	0.1431	0.040*	
N3	0.66090 (15)	0.51363 (15)	−0.07230 (14)	0.0329 (4)	
H3	0.5844	0.5345	−0.0898	0.039*	
N4	0.5646 (2)	0.74999 (18)	−0.58613 (18)	0.0558 (5)	
C11	0.7533 (2)	0.19656 (18)	−0.00942 (18)	0.0385 (4)	
H11	0.8616	0.2500	0.0263	0.046*	
C12	0.7254 (3)	0.1419 (2)	0.0981 (2)	0.0670 (7)	
H12A	0.6181	0.0940	0.0675	0.080*	
H12B	0.7635	0.2172	0.1799	0.080*	
C13	0.8027 (4)	0.0438 (3)	0.1304 (3)	0.0905 (10)	
H13A	0.9108	0.0945	0.1720	0.109*	
H13B	0.7765	0.0044	0.1949	0.109*	
C14	0.7562 (3)	−0.0698 (2)	0.0036 (3)	0.0779 (8)	
H14A	0.8118	−0.1262	0.0263	0.093*	
H14B	0.6500	−0.1271	−0.0324	0.093*	
C15	0.7858 (3)	−0.0128 (3)	−0.1009 (3)	0.0711 (7)	
H15A	0.7513	−0.0870	−0.1825	0.085*	
H15B	0.8931	0.0377	−0.0678	0.085*	
C16	0.7059 (3)	0.0814 (2)	−0.1346 (2)	0.0537 (6)	
H16A	0.7290	0.1189	−0.2012	0.064*	
H16B	0.5981	0.0292	−0.1739	0.064*	
C21	0.9318 (2)	0.61724 (18)	0.27734 (18)	0.0372 (4)	
H21	0.8543	0.6058	0.3101	0.045*	
C22	0.9661 (3)	0.7503 (2)	0.2469 (2)	0.0772 (8)	
H22A	0.8738	0.7497	0.1823	0.093*	
H22B	1.0344	0.7586	0.2051	0.093*	
C23	1.0354 (4)	0.8726 (3)	0.3722 (3)	0.0995 (10)	
H23A	1.0617	0.9557	0.3479	0.119*	
H23B	0.9625	0.8708	0.4078	0.119*	
C24	1.1732 (3)	0.8739 (3)	0.4796 (3)	0.0952 (11)	
H24A	1.2118	0.9505	0.5605	0.114*	
H24B	1.2503	0.8853	0.4477	0.114*	
C25	1.1371 (3)	0.7418 (3)	0.5140 (2)	0.0760 (8)	
H25A	1.0672	0.7350	0.5540	0.091*	
H25B	1.2284	0.7426	0.5803	0.091*	
C26	1.0679 (2)	0.6172 (2)	0.3871 (2)	0.0556 (6)	
H26A	1.1422	0.6182	0.3533	0.067*	
H26B	1.0395	0.5342	0.4110	0.067*	
C30	0.74698 (18)	0.54829 (18)	−0.13909 (17)	0.0333 (4)	
C31	0.69513 (18)	0.60806 (17)	−0.25097 (17)	0.0314 (4)	
C32	0.79254 (19)	0.65206 (19)	−0.30874 (19)	0.0399 (5)	
H32	0.8865	0.6487	−0.2739	0.048*	
C33	0.7513 (2)	0.7005 (2)	−0.41680 (19)	0.0434 (5)	
H33	0.8163	0.7296	−0.4556	0.052*	
C34	0.6122 (2)	0.70486 (19)	−0.46623 (17)	0.0396 (5)	
C35	0.5142 (2)	0.6639 (2)	−0.41019 (19)	0.0426 (5)	
H35	0.4210	0.6688	−0.4449	0.051*	
C36	0.55611 (19)	0.61538 (19)	−0.30166 (18)	0.0387 (4)	
H36	0.4911	0.5876	−0.2625	0.046*	

### Atomic displacement parameters (Å^2^)

**Table:**

	*U*^11^	*U*^22^	*U*^33^	*U*^12^	*U*^13^	*U*^23^
P	0.0300 (3)	0.0420 (3)	0.0339 (3)	0.0222 (2)	0.0166 (2)	0.0181 (2)
O1	0.0394 (7)	0.0653 (9)	0.0482 (8)	0.0331 (6)	0.0286 (6)	0.0328 (7)
O2	0.0414 (7)	0.0855 (11)	0.0506 (8)	0.0435 (7)	0.0294 (7)	0.0358 (8)
O3	0.0942 (13)	0.1094 (15)	0.0620 (11)	0.0376 (11)	0.0452 (11)	0.0528 (11)
O4	0.0850 (13)	0.159 (2)	0.0865 (13)	0.0732 (13)	0.0370 (11)	0.0858 (14)
N1	0.0420 (8)	0.0447 (9)	0.0346 (8)	0.0248 (7)	0.0071 (7)	0.0117 (7)
N2	0.0320 (8)	0.0443 (9)	0.0326 (8)	0.0249 (7)	0.0149 (7)	0.0108 (7)
N3	0.0298 (7)	0.0472 (9)	0.0370 (8)	0.0261 (7)	0.0186 (7)	0.0207 (7)
N4	0.0583 (11)	0.0567 (12)	0.0434 (10)	0.0178 (9)	0.0139 (9)	0.0231 (9)
C11	0.0371 (9)	0.0358 (10)	0.0421 (10)	0.0194 (8)	0.0116 (9)	0.0146 (9)
C12	0.1058 (19)	0.0580 (14)	0.0472 (13)	0.0402 (14)	0.0349 (13)	0.0232 (12)
C13	0.140 (3)	0.0675 (17)	0.0624 (16)	0.0553 (17)	0.0237 (17)	0.0380 (15)
C14	0.0989 (19)	0.0490 (14)	0.0778 (17)	0.0416 (14)	0.0189 (16)	0.0225 (14)
C15	0.0883 (18)	0.0575 (15)	0.0794 (17)	0.0467 (14)	0.0356 (15)	0.0188 (14)
C16	0.0703 (14)	0.0544 (13)	0.0483 (12)	0.0356 (11)	0.0280 (11)	0.0199 (11)
C21	0.0390 (10)	0.0445 (11)	0.0358 (10)	0.0222 (8)	0.0204 (8)	0.0114 (9)
C22	0.115 (2)	0.0475 (14)	0.0572 (15)	0.0301 (14)	0.0266 (15)	0.0183 (12)
C23	0.140 (3)	0.0415 (15)	0.088 (2)	0.0229 (16)	0.036 (2)	0.0070 (15)
C24	0.090 (2)	0.069 (2)	0.083 (2)	−0.0029 (16)	0.0424 (18)	−0.0226 (17)
C25	0.0643 (15)	0.105 (2)	0.0405 (13)	0.0401 (15)	0.0111 (12)	−0.0041 (14)
C26	0.0570 (13)	0.0683 (15)	0.0364 (11)	0.0329 (11)	0.0125 (10)	0.0075 (11)
C30	0.0309 (9)	0.0421 (10)	0.0314 (9)	0.0196 (8)	0.0145 (8)	0.0107 (8)
C31	0.0305 (9)	0.0338 (9)	0.0306 (9)	0.0141 (7)	0.0142 (8)	0.0081 (8)
C32	0.0320 (9)	0.0478 (11)	0.0426 (11)	0.0169 (8)	0.0175 (8)	0.0168 (9)
C33	0.0419 (10)	0.0517 (12)	0.0413 (11)	0.0162 (9)	0.0235 (9)	0.0187 (10)
C34	0.0466 (11)	0.0377 (10)	0.0287 (9)	0.0137 (8)	0.0124 (9)	0.0120 (8)
C35	0.0360 (10)	0.0548 (12)	0.0412 (11)	0.0238 (9)	0.0141 (9)	0.0209 (10)
C36	0.0351 (9)	0.0524 (12)	0.0390 (10)	0.0224 (9)	0.0204 (9)	0.0205 (9)

### Geometric parameters (Å, °)

**Table:**

P—O1	1.4739 (13)	C16—H16B	0.9700
P—N1	1.6174 (16)	C21—C22	1.502 (3)
P—N2	1.6233 (14)	C21—C26	1.505 (3)
P—N3	1.6966 (14)	C21—H21	0.9800
O2—C30	1.222 (2)	C22—C23	1.510 (3)
O3—N4	1.210 (2)	C22—H22A	0.9700
O4—N4	1.206 (2)	C22—H22B	0.9700
N1—C11	1.468 (2)	C23—C24	1.498 (4)
N1—H1	0.8600	C23—H23A	0.9700
N2—C21	1.473 (2)	C23—H23B	0.9700
N2—H2	0.8600	C24—C25	1.514 (4)
N3—C30	1.362 (2)	C24—H24A	0.9700
N3—H3	0.8600	C24—H24B	0.9700
N4—C34	1.471 (2)	C25—C26	1.536 (3)
C11—C12	1.498 (3)	C25—H25A	0.9700
C11—C16	1.509 (3)	C25—H25B	0.9700
C11—H11	0.9800	C26—H26A	0.9700
C12—C13	1.538 (3)	C26—H26B	0.9700
C12—H12A	0.9700	C30—C31	1.507 (2)
C12—H12B	0.9700	C31—C36	1.387 (2)
C13—C14	1.514 (4)	C31—C32	1.391 (2)
C13—H13A	0.9700	C32—C33	1.374 (3)
C13—H13B	0.9700	C32—H32	0.9300
C14—C15	1.485 (4)	C33—C34	1.373 (3)
C14—H14A	0.9700	C33—H33	0.9300
C14—H14B	0.9700	C34—C35	1.375 (3)
C15—C16	1.521 (3)	C35—C36	1.380 (3)
C15—H15A	0.9700	C35—H35	0.9300
C15—H15B	0.9700	C36—H36	0.9300
C16—H16A	0.9700		
			
O1—P—N1	119.46 (8)	C22—C21—C26	111.50 (17)
O1—P—N2	111.42 (7)	N2—C21—H21	107.9
N1—P—N2	105.05 (8)	C22—C21—H21	107.9
O1—P—N3	105.89 (7)	C26—C21—H21	107.9
N1—P—N3	101.89 (8)	C21—C22—C23	112.7 (2)
N2—P—N3	112.93 (7)	C21—C22—H22A	109.0
C11—N1—P	129.49 (13)	C23—C22—H22A	109.0
C11—N1—H1	115.3	C21—C22—H22B	109.0
P—N1—H1	115.3	C23—C22—H22B	109.0
C21—N2—P	122.67 (11)	H22A—C22—H22B	107.8
C21—N2—H2	118.7	C24—C23—C22	111.5 (3)
P—N2—H2	118.7	C24—C23—H23A	109.3
C30—N3—P	122.74 (11)	C22—C23—H23A	109.3
C30—N3—H3	118.6	C24—C23—H23B	109.3
P—N3—H3	118.6	C22—C23—H23B	109.3
O4—N4—O3	122.9 (2)	H23A—C23—H23B	108.0
O4—N4—C34	118.1 (2)	C23—C24—C25	110.5 (2)
O3—N4—C34	118.90 (19)	C23—C24—H24A	109.6
N1—C11—C12	112.89 (17)	C25—C24—H24A	109.6
N1—C11—C16	109.24 (15)	C23—C24—H24B	109.6
C12—C11—C16	110.75 (16)	C25—C24—H24B	109.6
N1—C11—H11	107.9	H24A—C24—H24B	108.1
C12—C11—H11	107.9	C24—C25—C26	111.3 (2)
C16—C11—H11	107.9	C24—C25—H25A	109.4
C11—C12—C13	111.0 (2)	C26—C25—H25A	109.4
C11—C12—H12A	109.4	C24—C25—H25B	109.4
C13—C12—H12A	109.4	C26—C25—H25B	109.4
C11—C12—H12B	109.4	H25A—C25—H25B	108.0
C13—C12—H12B	109.4	C21—C26—C25	111.20 (18)
H12A—C12—H12B	108.0	C21—C26—H26A	109.4
C14—C13—C12	111.5 (2)	C25—C26—H26A	109.4
C14—C13—H13A	109.3	C21—C26—H26B	109.4
C12—C13—H13A	109.3	C25—C26—H26B	109.4
C14—C13—H13B	109.3	H26A—C26—H26B	108.0
C12—C13—H13B	109.3	O2—C30—N3	121.79 (16)
H13A—C13—H13B	108.0	O2—C30—C31	119.65 (16)
C15—C14—C13	110.7 (2)	N3—C30—C31	118.55 (14)
C15—C14—H14A	109.5	C36—C31—C32	119.53 (17)
C13—C14—H14A	109.5	C36—C31—C30	123.97 (16)
C15—C14—H14B	109.5	C32—C31—C30	116.44 (15)
C13—C14—H14B	109.5	C33—C32—C31	120.64 (17)
H14A—C14—H14B	108.1	C33—C32—H32	119.7
C14—C15—C16	111.0 (2)	C31—C32—H32	119.7
C14—C15—H15A	109.4	C34—C33—C32	118.64 (18)
C16—C15—H15A	109.4	C34—C33—H33	120.7
C14—C15—H15B	109.4	C32—C33—H33	120.7
C16—C15—H15B	109.4	C33—C34—C35	122.12 (17)
H15A—C15—H15B	108.0	C33—C34—N4	118.82 (18)
C11—C16—C15	111.21 (18)	C35—C34—N4	119.03 (17)
C11—C16—H16A	109.4	C34—C35—C36	119.04 (17)
C15—C16—H16A	109.4	C34—C35—H35	120.5
C11—C16—H16B	109.4	C36—C35—H35	120.5
C15—C16—H16B	109.4	C35—C36—C31	120.01 (17)
H16A—C16—H16B	108.0	C35—C36—H36	120.0
N2—C21—C22	112.00 (16)	C31—C36—H36	120.0
N2—C21—C26	109.51 (15)		
			
O1—P—N1—C11	−95.68 (17)	C23—C24—C25—C26	56.3 (3)
N2—P—N1—C11	30.22 (19)	N2—C21—C26—C25	177.50 (18)
N3—P—N1—C11	148.19 (16)	C22—C21—C26—C25	53.0 (3)
O1—P—N2—C21	−35.25 (16)	C24—C25—C26—C21	−55.1 (3)
N1—P—N2—C21	−166.00 (13)	P—N3—C30—O2	−4.9 (2)
N3—P—N2—C21	83.79 (15)	P—N3—C30—C31	173.61 (12)
O1—P—N3—C30	170.69 (13)	O2—C30—C31—C36	170.60 (17)
N1—P—N3—C30	−63.67 (15)	N3—C30—C31—C36	−8.0 (3)
N2—P—N3—C30	48.50 (15)	O2—C30—C31—C32	−6.5 (2)
P—N1—C11—C12	62.5 (2)	N3—C30—C31—C32	174.97 (15)
P—N1—C11—C16	−173.78 (14)	C36—C31—C32—C33	−1.1 (3)
N1—C11—C12—C13	177.69 (19)	C30—C31—C32—C33	176.11 (16)
C16—C11—C12—C13	54.8 (3)	C31—C32—C33—C34	0.2 (3)
C11—C12—C13—C14	−54.6 (3)	C32—C33—C34—C35	0.7 (3)
C12—C13—C14—C15	55.3 (3)	C32—C33—C34—N4	−176.94 (17)
C13—C14—C15—C16	−56.7 (3)	O4—N4—C34—C33	−174.8 (2)
N1—C11—C16—C15	178.52 (19)	O3—N4—C34—C33	7.5 (3)
C12—C11—C16—C15	−56.5 (3)	O4—N4—C34—C35	7.5 (3)
C14—C15—C16—C11	57.7 (3)	O3—N4—C34—C35	−170.23 (19)
P—N2—C21—C22	−81.3 (2)	C33—C34—C35—C36	−0.7 (3)
P—N2—C21—C26	154.44 (15)	N4—C34—C35—C36	176.95 (17)
N2—C21—C22—C23	−176.5 (2)	C34—C35—C36—C31	−0.2 (3)
C26—C21—C22—C23	−53.4 (3)	C32—C31—C36—C35	1.1 (3)
C21—C22—C23—C24	55.0 (4)	C30—C31—C36—C35	−175.88 (17)
C22—C23—C24—C25	−55.9 (3)		

### Hydrogen-bond geometry (Å, °)

**Table:**

*D*—H···*A*	*D*—H	H···*A*	*D*···*A*	*D*—H···*A*
N1—H1···O4^i^	0.86	2.54	3.305 (2)	148
N2—H2···O2^ii^	0.86	2.25	3.0578 (18)	156
N3—H3···O1^iii^	0.86	1.97	2.8229 (18)	170

Symmetry codes: (i) −*x*+1, −*y*+1, −*z*−1; (ii) −*x*+2, −*y*+1, −*z*; (iii) −*x*+1, −*y*+1, −*z*.
